# Whole-Genome Sequence of an African Swine Fever Virus Isolate from the Czech Republic

**DOI:** 10.1128/MRA.00948-20

**Published:** 2020-10-29

**Authors:** Jan H. Forth, Leonie F. Forth, Petr Václavek, Pavel Barták, Dirk Höper, Martin Beer, Sandra Blome

**Affiliations:** a Friedrich-Loeffler-Institut, Federal Research Institute for Animal Health, Greifswald, Germany; b State Veterinary Institute Jihlava, Jihlava, Czech Republic; KU Leuven

## Abstract

Between June 2017 and April 2018, an outbreak of African swine fever (ASF) affected wild boar in the southeast of the Czech Republic. Here, we present the whole-genome sequence of the causative ASF virus. It belongs to genotype II and shows very high identity with other strains from Eastern Europe.

## ANNOUNCEMENT

African swine fever (ASF) is a hemorrhagic disease of domestic pigs and European wild boar (1). In 2007, its causative agent, African swine fever virus (ASFV), a DNA virus with a double-stranded genome of 170 to 194 kbp (1), emerged in eastern Europe (The Republic of Georgia) (2). Since then, ASFV has spread through neighboring countries, reaching the European Union in 2014 and western Europe as well as Asia in 2018 (2, 3).

From June 2017 to April 2018, an outbreak was recorded in the Czech Republic. The first case was detected in an urban area in the cadastral territory Příluky u Zlína, Zlín District, Zlín Region, followed by 229 cases of ASFV-positive wild boar that were found dead (or hunted) until 15 April 2018 in an area of approximately 1,033 km^2^ around Zlín (OIE-WAHIS Interface, https://www.oie.int/wahis_2/public/wahid.php/Diseaseinformation/Diseaseoutbreakmaps, accessed 12 August 2019).

In July 2017, a spleen sample of an ASFV-positive wild boar was sent from the Czech National Reference Laboratory for ASF, the State Veterinary Institute Jihlava, to the German Friedrich-Loeffler-Institut for whole-genome sequencing.

The sample was homogenized in 200 μl phosphate-buffered saline (PBS) with two 5-mm stainless steel beads in a TissueLyser II (Qiagen) for 3 min at 30 hz. After centrifugation at 10,000 × *g* for 3 min, DNA was extracted from the supernatant using the High Pure template preparation kit (Roche) according to the manufacturer’s instructions. Subsequently, an Illumina-compatible library was prepared using the GeneRead DNA library I core kit (Qiagen) and NEXTflex dual-index DNA barcodes 1 to 96 (Bioo Scientific), quality checked using a bioanalyzer (Agilent) and the KAPA library quantification kit (Roche) as described elsewhere (4, 5), and sequenced on an Illumina MiSeq instrument in 300-bp paired-end mode.

In total, 4,694,442 reads were produced, quality trimmed, and mapped against the ASFV Georgia 2007/1 sequence (International Nucleotide Sequence Database Collaboration [INSDC]/ENA accession no. FR682468.2) as a reference (6) using the Newbler v3.0 software (Roche) with default parameters, followed by subsequent *de novo* assembly of all mapped reads using SPAdes 3.11.0 with default parameters and automatically chosen k-mer sizes of 21, 33, 55, 77, 99, and 127 (7). The analysis resulted in 179,118 ASFV-specific reads (3.8% of the entire data set), of which 76,551 reads (1.63%) were unique and allowed for whole-genome assembly with a mean coverage of 118×. The assembled genome of ASFV CzechRepublic 2017/1 (LR722600.1) has a length of 190,595 bp, a G+C content of 38.4%, and inverted terminal repeat regions at the genome ends identical to those of the most complete ASFV genotype II sequence available, namely, ASFV Georgia 2007/1 (FR682468.2). Furthermore, it shows over 99.9% identity to all other available eastern European ASFV genotype II wild-type whole-genome sequences at INSDC databases visited on 12 August 2019 (using MAFFT v7.388 in Geneious 2019.2.3 with default parameters). Therefore, it unfortunately does not allow for any reliable conclusions regarding phylogenetic or geographic relationships.

However, we identified 23 differences in single nucleotides and a tandem repeat insertion in a previously known variable region (8) compared with ASFV Georgia 2007/1 (INSDC/ENA accession no. FR682468.2) (6) and ASFV Belgium 2018/1 (LR536725.1) (9). While 22 of these differences are located in homopolymer regions of up to 19 C nucleotides (where even modern sequencing platforms, such as the Illumina MiSeq, do not allow for discrimination between artifact and natural variability), 1 nucleotide transition at position 141519 was found to be specific for the ASFV CzechRepublic 2017/1 genome. Although this mutation is nonsynonymous (alanine1073valine) in the D1133L-ORF (a member helicase superfamily II and putative transcription factor) (1), no conclusions can be drawn on a change in virulence or virus attenuation in the absence of observations from the field or experimental biological characterization.

In conclusion, we are convinced that only whole-genome sequences, together with harmonized protocols and data sharing, can serve as a basis for the identification of new genetic markers that are needed for molecular epidemiology approaches and therefore are of utmost importance in the fight against ASF.

### Data availability.

This whole-genome shotgun project has been deposited in INSDC/ENA under the study accession no. PRJEB34070 and accession no. LR722600. The version described in this paper is the first version, LR722600.1.
